# Socio-economic differences in the uptake of HIV testing and associated factors in South Africa

**DOI:** 10.1186/s12889-021-11583-1

**Published:** 2021-08-26

**Authors:** Sean Jooste, Musawenkosi Mabaso, Myra Taylor, Alicia North, Yolande Shean, Leickness Chisamu Simbayi

**Affiliations:** 1 Human and Social Capabilities Research Division, Human Sciences Research Council, 118 Buitengracht St, Cape Town City Centre, Cape Town, 8000 South Africa; 2 University of KwaZulu-Natal, School of Nursing and Public Health, 238 Mazisi Kunene Rd, Glenwood, Durban, 4041 South Africa; 3 Deputy CEO for Research, Human Sciences Research Council, 118 Buitengracht St, Cape Town City Centre, Cape Town, 8000 South Africa; 4 Department of Psychiatry & Mental Health, University of Cape Town, Groote Schuur Dr, Observatory, Cape Town, 7700 South Africa

**Keywords:** UNAIDS 90–90-90 targets, HIV testing, Socio-economic status, South Africa

## Abstract

**Background:**

Improved understanding of barriers to HIV testing is important for reaching the first of the UNAIDS 90–90-90 targets, which states that 90% of HIV positive individuals ought to know their HIV status. This study examined socio-economic status (SES) differences in HIV testing uptake and associated factors among youth and adults 15 years and older in South Africa.

**Methods:**

This study used data from a national cross-sectional, population-based household survey conducted in 2017 using a multi-stage sampling design. A composite SES score was created using multiple correspondence analyses of household assets; households were classified into wealth quintiles and dichotomised into low SES/poorest (lowest 3 quintiles) and high SES/less-poor (highest 2 quintiles). Bivariate and multivariate logistic regression models were used to examine factors associated with the uptake of HIV testing in low and high SES households.

**Results:**

HIV testing uptake was 73.8 and 76.7% among low and high SES households, respectively, both of which were below the first 90 targets. Among both low and high SES households, increased HIV testing uptake was significantly associated with females than males. The decreased likelihood was significantly associated with residing in rural formal areas than urban areas, those with no education or low levels of educational attainment and alcohol drinkers among low SES households. Whites and Indians/Asians had a decreased likelihood than Black Africans in high SES households.

**Conclusions:**

HIV testing interventions should target males, residents in rural formal areas, those with no or low education and those that consume alcohol in low SES households, including Whites and Indians/Asians from high SES households in order to bridge socio-economic disparities in the uptake of HIV testing. This should entail expanding HIV testing beyond traditional centres for voluntary counselling and testing through outreach efforts, including mobile testing and home-based testing.

## Background

Sub-Saharan Africa (SSA) bears the largest burden of the HIV epidemic, with 53% of the world’s 36.9 million [31.1–43.9 million] people living with HIV in 2018 [1]. The HIV epidemic in South Africa is the largest globally, with a national prevalence of 14%, which translated to an estimated 7.9 million people living with HIV in 2017 [2]. HIV testing uptake serves as the foundation for the Joint United Nations Programme on HIV/AIDS (UNAIDS) strategic framework in the fight against HIV [3]. This framework specifies that 90% of HIV-positive people should be aware of their status, 90% of those diagnosed should receive sustained antiretroviral therapy (ART), and 90% of those on ART achieve viral suppression [3]. This framework was adopted in December 2014 by the South African government as the basis of its national response to the HIV/AIDS epidemic in the country. Achieving the UNAIDS and South African government targets hinges on reaching the key goal of identifying 90% of people living with HIV in order to start the treatment continuum.

HIV testing is crucial as it provides a diagnosis for people living with HIV for linkage to care and treatment [4]. Globally, progress is being made, and at the end of 2017, three-quarters of people living with HIV knew their status [1]. Despite the excellent strides made with HIV testing in SSA, awareness of HIV status remains lower than the rest of the world. Findings from 10 population-based studies in 2012 identified that the average percentage of people living with HIV who know their status was below 40% [5]. Several barriers to HIV testing have been identified in studies that have been done in SSA. In the 2005 Demographic and Health Survey in Cote d’Ivoire, a low socio-economic status was related to lower proportions of individuals testing for HIV testing [6, 7]. A multi-country study in Africa found that an increased probability of HIV testing was associated with higher SES [8]. In South Africa, a nationally representative survey found lower HIV testing among people with lower SES [9].

Various socio-economic factors impact HIV testing. For example, educational attainment, employment and income generation have been associated with increased uptake of testing through good access to HIV information and greater control over the decision to test [6–8, 10]. On the other hand, rural settings with widespread poverty have been associated with low HIV testing mainly due to insufficient HIV awareness and knowledge, and logistical constraints such as long travelling distances and affordability of transportation, which lead to poor access to health services and HIV testing [10–12]. Other barriers include lower risk perception of HIV infection [5, 7, 9, 13], fear of HIV status and stigma [13–15]. These barriers to testing will impede the goal of reaching viral suppression among people living with HIV [3]. Improved understanding of SES related testing barriers may be essential for designing interventions focused on reaching the first 90 targets.

Evidence shows that socio-economic status harms people’s wellbeing, especially in settings with widespread poverty and vast income inequality [16]. However, SES is a complex composite measure that typically incorporates social, economic and employment status and is measured by education, income and occupation, respectively [17]. The level of educational attainment may be associated with the type of employment/occupation, better living conditions and health care [8, 18]. Hence, educational level and inter-related socio-economic measures can sometimes show a weaker or stronger association with deprivation, resulting in a differential relationship with health outcomes than other indices such as wealth and income [17, 19–21].

Income has been the preferred unit of welfare analysis because it is directly comparable among observations, making it straightforward to interpret and use in the quantitative analysis [22, 23]. In the absence of income data, the asset index method is frequently used in many developing countries to complement income measures wealth [24].

This study examines the socio-economic differences in HIV testing uptake and associated factors using data from the 2017 South African national survey in. This survey did not collect data on household income, and this paper used an asset-based measure of SES.

## Methods

### Data

This study used data from the South African HIV household survey carried out in 2017. The details about the design and sampling of this national survey are given elsewhere [2]. Briefly, a multi-stage stratified random sampling approach was used for selecting residential households within small area layers from a national sampling frame developed by the national statistical agency [25].

Fieldworkers collected data and blood samples from consenting individuals using age-appropriate structured questionnaires [2]. This secondary data analysis focused on respondents 15 years and older who responded to the question on HIV testing. This group has been identified as a priority target for HIV testing services, treatment, and viral load suppression, including HIV prevention efforts.

### Primary outcome variable

The dependent primary outcome variable ‘HIV testing’ was defined as having accessed HIV testing services at least once before the survey.

The primary outcome variable was stratified by socio-economic status, which a composite index was constructed using multiple correspondence analyses (MCA), a data reduction technique for categorical data, based on questions about the presence or absence of basic services and ownership of household assets a binary indicator [22, 23]. These included ownership of a range of assets (radio, television, landline telephone, washing machine, refrigerator, personal computer/laptop/tablet, solar panel, motor vehicle), housing characteristics (the main source of energy for cooking) and access to basic services (source of drinking water, sanitation facilities, and electricity).

The indicators of asset ownership were organised into a matrix and each asset indicator was decomposed into a set of binary [23]. Then a household composite indicator score was computed by adding up all the weighted responses. The calculation of the household’s asset index score can is presented elsewhere [24]. The predicted score for each household was used to compute five wealth quintiles, which were then dichotomised into low SES (lowest 3 quintiles) and high SES (highest 2 quintiles) [26].

### Explanatory variables

 Socio-demographic variables included age groups (15–24 years, 25–49 years, and 50 years and older) sex (male and female), racial categories (Black African, White, Coloured, and Indian/Asian), and current marital status (married and not married; which included divorced/separated and widowed/widow), locality type (urban areas formal, rural informal areas, rural formal). Additional variables included the highest educational level completed (no education, primary, secondary, and tertiary) and current employment status (not employed and employed).

Behavioural variables included the age of first sex (having had sex either before or after 15 years of age), age of sexual partner (partner older by 5 years, partner younger by 5 years, a partner within 5 years), number of sexual partners in the last 12 months (one partner, and two or more sexual partners), condom use at last sex (no and yes), alcohol use risk score (abstainers, low, high, and hazardous risk drinkers) based on the Alcohol Use Disorder Identification Test (AUDIT) scale [27, 28], correct HIV knowledge and myth rejection (no and yes) based on responses from the following questions (Can AIDS be cured? Can a person reduce the risk of HIV by having fewer sexual partners? Can a healthy-looking person have HIV? Can a person get HIV by sharing food with someone who is infected? Can a person reduce the risk of getting HIV by using a condom every time he/she has sex?), self-perceived risk of contracting HIV infection (no and yes).

### Statistical analysis

All statistical analysis was done using STATA 15.0 (Stata Corporation, College Station, Texas, USA) software. Pearson’s chi-square test was used to compare differences between categorical variables. Comparison of differences in HIV testing between high and low SES in each categorical variable was assessed using a test for two proportions. Bivariate and multivariate logistic regression models were used to examine factors associated with the uptake of HIV testing. The analysis was stratified by asset-based SES yielding two models (low SES model and high SES model). Crude and adjusted odds ratios (aORs) with 95% confidence intervals (CI) and *p*-values less than 0.05 were reported for all statistically significant associations. Coefficient plots were used to display the results of the final models [29].

## Results

### Background characteristics of the study sample

Table 1 shows that over half of the sample was aged 25–49 years (53.5%) and female (52.1%). The majority were Black African (79.0%), not married (70.8%), had completed secondary education (67.6%) and resided in urban areas (69.5%). There were significant differences in characteristics between participants with low and high socio-economic status regarding age, race, marital status, level of education, employment status, and locality type (*p* < 0.001).

**Table 1:** Background characteristics of the sample by socio-economic status (SES) among youth and adults 15 years and older, South Africa 2017

Variable	Overall sample		Low SES		High SES		
	n	%	n	%	n	%	***p***-values
**Overall sample**	21,075		8504		12,571		
**Age categories**							
15–19 years	2762	11.5	1242	12.3	1520	10.8	< 0.001
20–24 years	2578	12.4	1209	14.3	1369	11.0	
25–49 years	9715	53.5	4021	54.9	5694	52.4	
50+ years	6020	22.6	2032	18.6	3988	25.8	
**Sex**							
Male	8812	47.9	3556	47.6	5256	48.2	0.481
Female	12,263	52.1	4948	52.4	7315	51.8	
**Race**							
Black African	13,747	79.0	7551	95.2	6196	66.1	< 0.001
White	1509	9.3	24	0.4	1485	16.3	
Coloured	3805	8.9	875	4.2	2930	12.6	
Indian/Asian	2014	2.9	54	0.2	1960	5.0	
**Current marital status**							
Married	6758	29.2	1751	19.6	5007	36.8	< 0.001
Not married	14,312	70.8	6752	80.4	7560	63.2	
**Highest educational level obtained**							
No education/primary	3278	16.8	1874	24.9	1404	10.9	< 0.001
Secondary	10,263	67.6	3889	70.4	6374	65.5	
Tertiary	2276	15.6	201	4.7	2075	23.6	
**Employment status**							
Unemployed	13,432	63.8	6053	71.7	7379	57.6	< 0.001
Employed	7352	36.2	2355	28.3	4997	42.4	
**Locality type**							
Urban	13,810	69.5	3202	48.5	10,608	86.2	< 0.001
Rural informal (tribal areas)	4909	25.8	3650	44.8	1259	10.8	
Rural (farms)	2356	4.7	1652	6.7	704	3.1	

Table 2 shows socio-demographic characteristics and reported uptake of HIV testing among youth and adults aged 15 years and older by SES. Overall, people with a high SES reported significantly higher HIV testing uptake than those with a low SES, 76.7% vs 73.8% (*p* < 0.001). HIV testing uptake was significantly higher among people aged 25–49 years, males, Black Africans, the employed, and those residing in rural informal areas in high SES compared to low SES (all *p* < 0.001). HIV testing uptake was also higher significantly higher among those aged 50 years and older, not married, residing in urban and rural formal areas in high SES compared to low SES (all *p* < 0.05).

**Table 2:** Socio-demographic characteristics and HIV testing by socio-economic status (SES) among youth and adults 15 years and older, South Africa 2017

Variable	Total		Low SES		High SES		
	N	Tested	n	Tested	n	Tested	***p***-value
**Overall**	21,075	75.4	8504	73.8	12,571	76.7	< 0.001
**Age categories**							
15–19 years	2762	41.9	1242	42.0	1520	41.9	0.958
20–24 years	2578	74.1	1209	73.2	1369	75.1	0.271
25–49 years	9715	85.2	4021	83.0	5694	87.1	< 0.001
50+ years	6020	69.9	2032	68.2	3988	70.9	0.031
**Sex of respondent**							
Male	8812	71.2	3556	67.3	5256	74.2	< 0.001
Female	12,263	79.4	4948	79.8	7315	79.0	0.283
**Race**							
Black African	13,747	76.7	7551	74.0	6196	79.7	< 0.001
White	1509	69.7	24	64.5	1485	69.8	0.575
Coloured	3805	74.1	875	71.6	2930	74.7	0.067
Indian/A	2014	63.9	54	62.7	1960	64.0	0.844
**Current marital status**							
Married	6758	81.5	1751	80.5	5007	81.9	0.194
Not married	14,312	72.9	6752	72.2	7560	73.7	0.044
**Highest level of education obtained**							
No education/primary	3278	71.9	1874	72.6	1404	70.7	0.232
Secondary	10,263	80.4	3889	81.0	6374	80.0	0.216
Tertiary	2276	86.1	201	89.1	2075	85.7	0.185
**Employment status**							
Unemployed	13,432	70.7	6053	71.0	7379	70.5	0.526
Employed	7352	84.0	2355	81.3	4997	85.5	< 0.001
**Locality type**							
Urban areas	13,810	77.5	3202	78.7	10,608	77.0	0.044
Rural informal areas	4909	70.5	3650	69.3	1259	74.7	< 0.001
Rural formal areas	2356	71.1	1652	69.4	704	74.0	0.025

Table 3 shows HIV–related risk characteristics and reported uptake of HIV testing among youth and adults aged 15 years and older by asset-based socio-economic status. HIV testing uptake was significantly higher among those with high SES versus low SES households. It varied significantly by sexual activity, age of sexual partner, alcohol consumption, correct HIV knowledge and myth rejection, self-perceived risk of HIV infection and HIV serostatus (All at *p* < 0.05).

**Table 3:** HIV related risk characteristics and HIV testing by socio-economic status (SES) among youth and adults 15 years and older, South Africa 2017

Variable	Total		Low SES		High SES		
Sexual activity	N	Tested	n	Tested	n	Tested	***p***-value
Never had sex	3142	42.3	1368	41.0	1920	45.1	0.019
Had sex	15,741	81.2	6766	80.4	9895	82.1	0.006
**Sexual debut**							
Sex before the age of 15 years	324	67.4	169	65.6	173	69.5	0.441
Sex at 15 years and older	4859	57.2	2269	58.0	2710	57.7	0.831
**Age of sexual partner**							
Partner more than 5 years younger	1971	82.2	2344	82.3	3908	85.2	0.002
Partner within five years	5849	84.3	813	81.3	1179	83.2	0.274
Partner more than 5 years older	2308	89.8	1073	90.3	1414	90.0	0.804
**Number of sexual partners in the past 12 months**							
1 sexual partner	9289	85.2	3827	84.5	6024	85.7	0.102
2+ or more sexual partners	892	82.5	438	79.4	504	86.2	0.006
**Condom use at last sex in the past 12 months**							
No condom use	6564	84.9	2445	83.8	4535	85.4	0.075
Yes condom use	3541	85.0	1817	84.1	1919	86.3	0.058
**AUDIT Score**							
Abstainers	13,037	73.5	5974	72.9	7835	74.7	0.017
Low risk (1–7)	3537	77.9	1177	76.8	2532	78.7	0.193
Risky level (8–15)	1257	79.2	557	76.2	761	81.4	0.022
High risk/harmful (16–19)	209	74.9	113	68.8	112	81.5	0.028
High risk/hazardous (20+)	226	70.6	135	68.7	104	72.9	0.480
**Correct HIV knowledge and myth rejection**							
No knowledge	12,575	74.1	5760	73.0	7518	75.5	0.001
Yes knowledge	7337	77.1	2726	75.6	5034	78.6	0.003
**Self-perceived risk of HIV infection**							
No risk	15,921	72.3	6089	69.3	10,681	74.8	< 0.001
Yes risk	2378	79.4	1363	78.2	1186	80.8	0.105
**HIV serostatus**							
HIV Positive	2358	87.6	1449	85.9	909	90.0	0.003
HIV Negative	12,044	74.8	4889	72.7	7155	76.2	< 0.001

### Factors associated with uptake of HIV testing

**Fig. 1:**
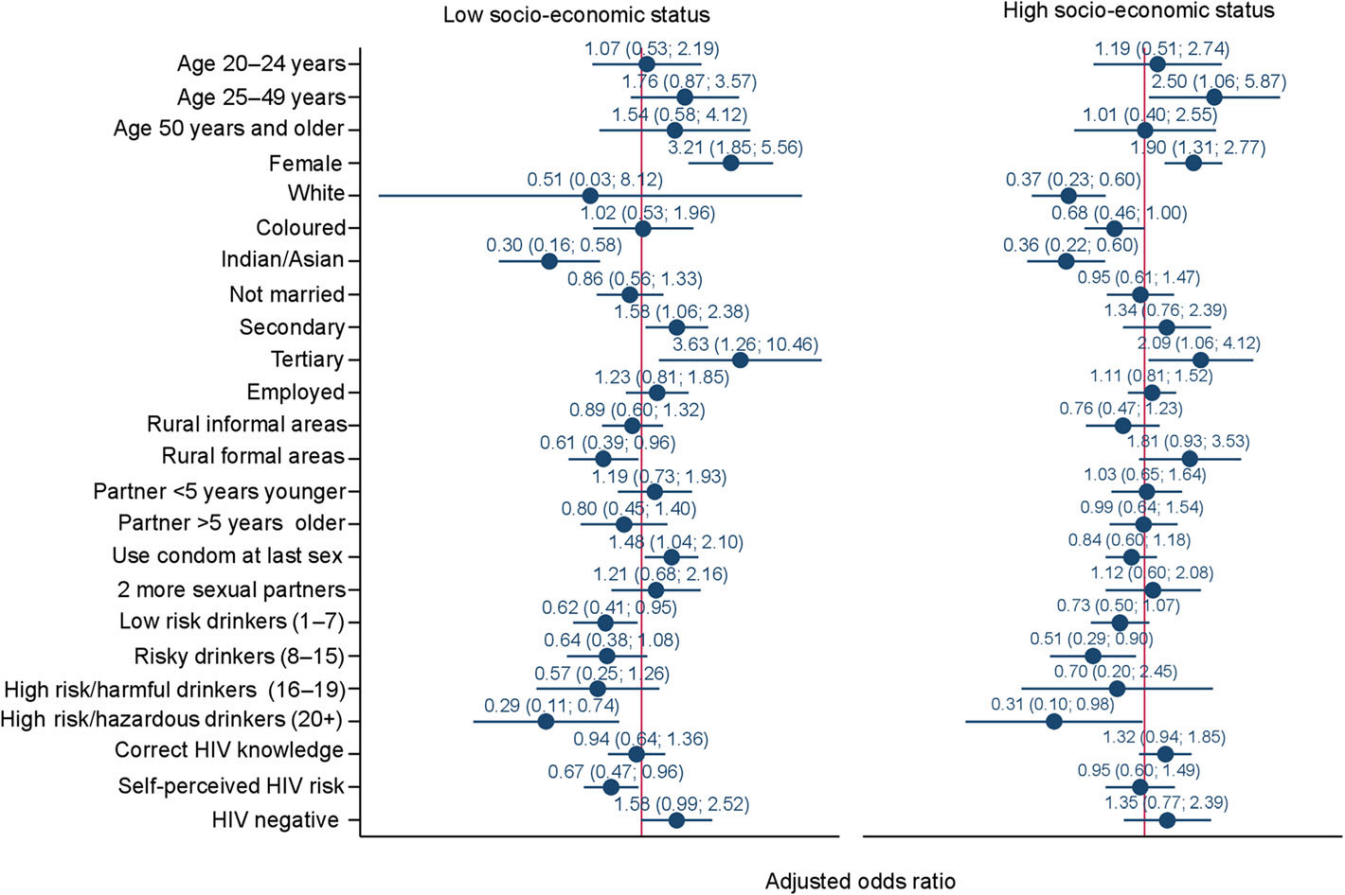
Multivariate models of factors associated with HIV testing by socio-economic status among youth and adults 15 years and older, South Africa 2017

Figure 1 shows the final adjusted models for multivariate logistic regression analysis of predictors of HIV testing uptake by asset-based socio-economic status among respondents aged 15 years and older. Among respondents from low SES households, females were significantly more likely to test for HIV [adjusted odds ratio (aOR) = 3.21, *p* < 0.001] than males. The increased likelihood of HIV testing uptake was significantly associated with respondents with secondary [aOR = 1.58, *p* = 0.026] and tertiary [aOR = 3.63, *p* = 0.017] level education, compared to those with no education or with primary level education completed. Those who used a condom at last sex were significantly more likely to test for HIV [aOR = 1.48, *p* = 0.028] compared to those who did not use a condom. The decreased likelihood of HIV testing uptake was significantly associated with respondents who engaged in low-risk drinking [OR = 0.62, *p* = 0.028 and high-risk drinking [OR = 0.29, *p* = 0.010] compared to those who abstained from alcohol. Respondents who perceived themselves as being at risk of HIV infection were also significantly less likely to test for HIV [OR = 0.67, *p* = 0.029] than their counterparts. The decreased likelihood of HIV testing uptake was significantly associated with respondents who resided in rural formal areas [OR = 0.61, *p* = 0.032] compared to those from urban areas.

Among respondents from high SES households, females were significantly more likely to test for HIV [aOR = 1.90, *p* < 0.001] than males. The increased likelihood of HIV testing uptake was significantly associated with respondents with tertiary [aOR = 2.09, *p* = 0.033] level education, compared to those with no education or with primary level education completed. The decreased likelihood of HIV testing uptake was significantly associated with being White [aOR = 0.37, *p* < 0.001], Coloured [aOR = 0.68, *p* = 0.052] and Indian/Asian [aOR = 0.08, *p* < 0.001] compared to being Black African. The decreased likelihood of HIV testing uptake was significantly associated with respondents who engaged in risky drinking [OR = 0.51, *p* = 0.019] and high-risk drinking [OR = 0.31, *p* = 0.046] compared to those who abstained from alcohol.

## Discussion

This secondary analysis of 2017 nationally representative population-based study showed that people with a high socio-economic status reported significantly higher HIV testing uptake than those with a low SES, 76.7% vs 73.8%. The differences in HIV testing between high and low SES were found in specific socio-demographic and HIV-related risk characteristics. HIV testing uptake was higher among those aged 25–49 years, males, Black Africans, the employed, and those residing in rural informal areas in high SES compared to low SES households. Furthermore, HIV testing uptake was higher among those aged 50 years and older, those not married, those residing in urban and rural formal areas in high SES compared to low SES households. In addition, HIV testing uptake was higher among high SES compared to low SES households and varied by sexual activity, age of sexual partner, alcohol consumption, correct HIV knowledge and myth rejection, self-perceived risk of HIV infection, and HIV serostatus. The observed differences occur against the background of policies and programs initiated to expand HIV testing by increasing the availability of quality HIV testing services (HTS) and its uptake in all public health facilities in South Africa [30, 31]. These findings highlight the importance of implementing diversified modes of HIV testing with tailored strategies to increase uptake among people characterised by low SES. Demographic and socio-economic predictors of HIV testing uptake are important for this tailored targeting of varied approaches among different population groups.

Findings from the final multivariate logistic regression models showed that among low SES households, there is a need to target males, those with no education or low levels of educational attainment, those residing in rural formal or tribal areas, low risk and high-risk drinkers and low self-perceived risk of HIV infection. The observed higher levels of HIV testing among females is consistent with global statistics [32, 33]. In South Africa, antenatal services are widely available, and women commonly receive HIV tests in this setting [30]. On the other hand, men are less likely than women to use health services and take an HIV test [34]. This limited male participation in HIV testing is worrisome since those HIV positive men who are unaware of their status may continue to engage in unsafe behaviour [35]. Research evidence shows that men can be encouraged to test for HIV by other men through door-to-door testing campaigns and health-care services targeted towards them [36]. HIV testing strategies that are more convenient and confidential, like community-based approaches and HIV self-testing, have increased HIV testing uptake among men [37, 38]. Other proposed strategies include social and cultural approaches that engage men as leaders (village chiefs and headmen) to promote an enabling environment to encourage health seeking behaviour and engagement in HTS [39].

Considering the evidence that educational attainment has been linked to increased HIV testing uptake [40–43], existing Government measures to strengthen and improve access to universal education among predominantly lower SES groups is key to addressing differential HIV testing patterns. Implementing the piloted provision of HTS in schools and during the further education and training phase is key in bridging HIV testing disparities in the country [44]. In addition, health promotion efforts should intensify education programs on HIV and make full utilisation of HIV testing and counselling services appealing to those with the least education or no formal education [45].

The finding that uptake of HIV testing was less likely among those in rural areas with low SES could be linked to limited resources and structural barriers to health care in terms of geographical and financial accessibility [45]. This suggests a need to expand HIV testing beyond traditional centres for voluntary counselling and testing through outreach efforts, including mobile testing and home-based testing in impoverished rural communities [46–49].

Congruent with current findings, alcohol consumption has also been a barrier to prior HIV testing in a population-based study [50]. Since alcohol use is particularly problematic in impoverished communities, it is important to increase awareness and knowledge of HIV among population groups who drink alcohol excessively and clients of addiction health services. Community-based HIV testing with facilitated linkage to care is also recommended for this group [51, 52]. These interventions should involve integrating substance and alcohol prevention components into national HIV awareness campaigns and screening and brief interventions for substance and alcohol use in HIV programs [52].

The association of low HIV testing uptake with a high self-perceived risk of HIV infection implies that individuals might refuse HIV testing even if they know that they are at high risk of HIV infection. This differs from the view that people who refuse HIV testing commonly do so because they do not perceive themselves to be at risk [53]. Nevertheless, awareness of HIV does not always translate to a perception of individual risk [54]. Self-perceived risk is an important factor in the uptake of HIV testing, and HIV testing campaigns should incorporate HIV risk perception assessments and interventions to correct false risk perception, encourage HIV testing uptake, and link individuals to care [55, 56].

The results from the high SES model showed that Whites and Indians/Asians were less likely to test for HIV than Black Africans. The observed racial differences in HIV testing can be attributed to the low self-perceived risk of HIV infection among minority groups. The promotion of HIV testing is an important component of primary and secondary HIV prevention strategies [30], yet little is known about the impact of missed opportunities for HIV testing and delayed presentation to HIV services among minority groups. There is, therefore, a need for population-based studies of HIV testing behaviours of minority groups in South Africa.

Consistent with other studies, our results found a positive association between HIV testing and condom use in low SES households [57, 58]. Other studies found poor condom use, whether tested or not tested for HIV, indicating no association between condom use and HIV testing [59, 60]. The current results are encouraging since condom use and uptake of HIV testing are key strategies for preventing HIV transmission. Despite the reported improvement in HIV testing uptake in the country, it is still unacceptably low among the youth and male population. Furthermore, consistent condom use remains relatively poor in the country [2]. Therefore, there is an urgent need for the National HIV programme to continue expanding efforts of scaling up HTS and promoting both correct and consistent condom use towards reducing HIV prevalence and incidence in South Africa.

The study has several limitations. The analysis is based on self–reported HIV testing and risk factors, which are prone to social desirability and recall bias. There has been some concern that the asset index has an urban bias as it is based on assets that capture social stratification better in urban than in rural settings, making rural asset households look poorer than they should [61, 62]. This suggests that the index may exaggerate the urban-rural differences. Furthermore, due to the cross-sectional design of the current study, causal inferences cannot be drawn, and the analysis is only limited to identifying associations. Despite these limitations, this study used a nationally representative sample, and the findings are generalisable to the entire country.

## Conclusions

This study revealed that socio-economic inequalities in the uptake of HIV testing remain substantial in the country, despite more than a decade of the increasing availability of quality HTS. Social-demographic factors and HIV-related risk factors have an influence on the differential uptake of HIV testing by SES. The findings suggest diverse and targeted modes of HIV testing with interventions at the different socio-demographic and socio-economic levels focusing on their specific testing barriers to bridge the socio-economic gap in the uptake of HIV testing towards reaching the first 90 of the 90–90-90 targets in South Africa.

## Data Availability

The datasets used and/or analysed during the current study are available from the corresponding author on reasonable request.

## Abbreviations

AIDS: Acquired Immune Deficiency Syndrome
ART: Antiretroviral therapy
CI: Confidence Interval
HIV: Human Immunodeficiency Virus
HTS: HIV testing services
MCA: Multiple correspondence analysis
SAL: Small area layer
SES: Socio-economic status
SSA: Sub-Saharan Africa
UNAIDS: Joint United Nations Programme on HIV/AIDS
VP: Visiting Point
